# A Survey of Deep Learning-Based Multimodal Emotion Recognition: Speech, Text, and Face

**DOI:** 10.3390/e25101440

**Published:** 2023-10-12

**Authors:** Hailun Lian, Cheng Lu, Sunan Li, Yan Zhao, Chuangao Tang, Yuan Zong

**Affiliations:** 1 Key Laboratory of Child Development and Learning Science (Ministry of Education), Southeast University, Nanjing 210000, China; lianhailun@seu.edu.cn (H.L.); cheng.lu@seu.edu.cn (C.L.); 230189473@seu.edu.cn (S.L.); zhaoyan@seu.edu.cn (Y.Z.); tcg2016@seu.edu.cn (C.T.); 2 School of Information Science and Engineering, Southeast University, Nanjing 210000, China; 3 School of Biological Science and Medical Engineering, Southeast University, Nanjing 210000, China

**Keywords:** deep learning, fusion method, multimodal emotion recognition, survey

## Abstract

Multimodal emotion recognition (MER) refers to the identification and understanding of human emotional states by combining different signals, including—but not limited to—text, speech, and face cues. MER plays a crucial role in the human–computer interaction (HCI) domain. With the recent progression of deep learning technologies and the increasing availability of multimodal datasets, the MER domain has witnessed considerable development, resulting in numerous significant research breakthroughs. However, a conspicuous absence of thorough and focused reviews on these deep learning-based MER achievements is observed. This survey aims to bridge this gap by providing a comprehensive overview of the recent advancements in MER based on deep learning. For an orderly exposition, this paper first outlines a meticulous analysis of the current multimodal datasets, emphasizing their advantages and constraints. Subsequently, we thoroughly scrutinize diverse methods for multimodal emotional feature extraction, highlighting the merits and demerits of each method. Moreover, we perform an exhaustive analysis of various MER algorithms, with particular focus on the model-agnostic fusion methods (including early fusion, late fusion, and hybrid fusion) and fusion based on intermediate layers of deep models (encompassing simple concatenation fusion, utterance-level interaction fusion, and fine-grained interaction fusion). We assess the strengths and weaknesses of these fusion strategies, providing guidance to researchers to help them select the most suitable techniques for their studies. In summary, this survey aims to provide a thorough and insightful review of the field of deep learning-based MER. It is intended as a valuable guide to aid researchers in furthering the evolution of this dynamic and impactful field.

## 1. Introduction

Emotion recognition is an important area of research because it enables computers to accurately comprehend human emotions and provide intelligent responses to meet human requirements. In educational settings, emotions can play a critical role in student performance and well-being. Emotion recognition technology can be utilized to monitor students’ emotional states, allowing educators to gain a deeper understanding of their academic progress and overall condition. Emotion recognition has potential in the healthcare sector as it can assist doctors in understanding the emotional states of their patients, thus enabling the provision of individualized and tailored medical services. Emotion recognition also has the capacity to enhance intelligent customer service systems by detecting the emotional needs of users and offering more personalized services. As a result, emotion recognition technologies have emerged as a significant research area in artificial intelligence, attracting a great deal of attention for their potential to revolutionize how humans and computers interact.

During the initial phases of emotion recognition research, researchers primarily concentrated on the recognition of single modalities, e.g., speech emotion recognition [1,2,3,4,5] text emotion recognition [6,7], and facial expression recognition [8,9,10,11]. However, the limited accuracy of single-modal emotional judgments caused by incomplete data and susceptibility to noise led to an increase in the utilization of multiple modalities by researchers in making emotional evaluations. In light of this, multimodal emotion recognition (MER) was developed. Moreover, MER can leverage the maximal mutual information to quantify the dependency between different modality features, extracting the most informative and discriminative features from each modality. This technique further enhances the model’s capacity to make accurate emotional distinctions. As such, MER has captured the attention of many researchers, aiming to combine information from various modalities, which can corroborate and complement each other, thus providing more comprehensive and accurate information for emotional judgment and significantly improving the performance of emotional judgments [12,13,14,15].

With the rapid development of deep learning techniques, MER based on deep learning has emerged as one of the most prominent research topics in the field of emotion recognition [16]. Central to this is the design of specific network architectures and associated loss functions. Many current designs of loss functions draw from principles related to entropy, with cross-entropy loss functions being a predominant example. Essentially, a vast array of deep learning architectures optimize their models by either maximizing or minimizing certain entropy-associated loss functions [17,18,19,20]. This flexibility suggests that deep learning techniques can be adeptly customized to harness the interactions between various modalities, thereby gleaning features rich in emotional information for accurate emotion identification. An increasing number of researchers are adopting deep learning methodologies for MER, yielding commendable outcomes. Given this burgeoning interest, a comprehensive survey of the most recent methodologies and a forecast of future research directions have become imperative. Gu et al. [21] provided a comprehensive analysis of the framework and research methods applicable to video and music emotion recognition, with particular emphasis on deep learning techniques. However, this survey is deficient in its detailed description of existing multimodal databases and feature specifications. Koromilas et al. [22] exhaustively introduced an intermediate fusion method based on a deep multimodal learning architecture, but failed to mention early fusion and late fusion techniques. Zhang et al. [23] thoroughly depicted single-modal emotion recognition technologies, such as facial expression recognition, speech emotion recognition, and text emotion recognition. For MER, only feature-level and decision-level fusion methods were summarized. Given these observations, it becomes clear that existing surveys have not fully encapsulated the advancements in the deep domain of MER. Consequently, there is an urgent need for a comprehensive survey article on deep multimodal emotion recognition, which will facilitate a well-rounded understanding of the latest technological developments in the field.

Our survey aims to fill the existing gap in the field of deep learning-based MER. Unlike existing surveys, our work offers a more comprehensive exploration of the deep learning-based MER domain. We delve not only into different fusion strategies (early fusion, late fusion, hybrid fusion, simple concatenation fusion, utterance-level interaction fusion, and fine-grained interaction fusion) but also provide an extensive analysis of multimodal datasets and emotional feature extraction methods. This comprehensive analysis sets our research apart from prior surveys, which often concentrated on singular aspects of MER. Specifically, we first introduce widely employed MER datasets, conducting a systematic analysis of their inherent characteristics and potential challenges. We also delineate the merits and demerits of these datasets, aiding researchers in selecting the most suitable datasets for their specific studies. Secondly, we meticulously explore various approaches to multimodal emotional feature extraction, emphasizing the strengths and weaknesses of each method. Finally, we provide a comprehensive analysis of MER algorithms, with a focus on early, late, hybrid, and intermediate layer fusion strategies. We evaluate the pros and cons of these fusion strategies, offering valuable insights to assist researchers in selecting the most appropriate fusion techniques. In summary, this survey offers a thorough and insightful comprehension of the present state of the art and future research avenues in the field of MER. We hope that our research will fill the current gap in the field and serve as a valuable reference for researchers aiming to acquire a comprehensive understanding of the most recent advancements and forthcoming research prospects in the realm of MER.

**Figure 1:**
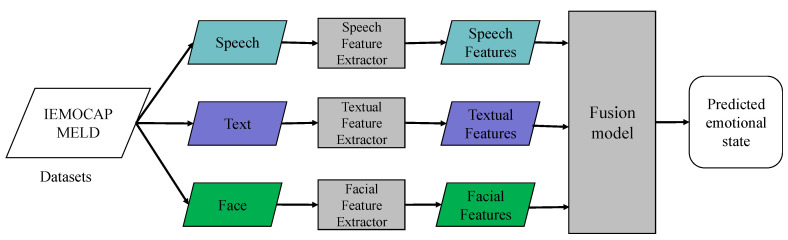
General framework of a typical multimodal emotion recognition system.

Figure 1 illustrates the comprehensive framework of a typical deep learning-based MER, which also delineates the structure of this survey. The organization of this paper is as follows: Section 2 presents widely used MER datasets and their characteristics and challenges. Section 3 delves into multimodal emotional feature extraction methods. Section 4 details MER algorithms and analyzes their advantages, disadvantages, and scope of application. Section 5 elucidates evaluation metrics prevalently employed in multimodal emotion recognition. Section 6 analyzes the results of existing research. Finally, Section 7 provides a summation of the paper’s main content and conclusions, highlighting future challenges in MER.

## 2. Popular Datasets in Multimodal Emotion Recognition

In the field of MER, several key datasets have been developed to facilitate research and experimental progress. While the existing literature presents a wide range of datasets, each with its unique advantages and limitations, there are still areas that warrant further attention. Here, we aim to provide an in-depth review of these existing resources. Table 1 presents the most popular datasets used for MER (including speech, text, and facial expression information).

### 2.1. IEMOCAP

The Interactive Emotional Dyadic Motion Capture (IEMOCAP) dataset, assembled by the Signal Analysis and Interpretation Lab (SAIL) at the University of Southern California, serves as a prominent multimodal resource for emotion recognition research [24]. The dataset amalgamates diverse modalities such as video, speech, facial motion capture, and text data, enabling comprehensive examination of emotional states in interactive circumstances. Ten actors, an equal mix of males and females, contributed to the data collection. These actors, paired by gender and divided into five groups, performed both scripted and improvised dialogues. This assortment of dialogues enriches the dataset with a variety of emotional content, enhancing its representativeness of real-world emotional communication. IEMOCAP comprises 4784 improvised and 5255 scripted conversations, providing a wide spectrum of emotional contexts for analysis. The dialogues span nine discrete emotions (happiness, sadness, anger, surprise, fear, disgust, frustration, excitement, and neutrality), in addition to continuous emotional dimensions like activation, arousal, and dominance, facilitating a more dynamic emotional analysis. IEMOCAP’s chief strength resides in its authenticity, as the emotions captured are natural, not simulated, thereby helping deep learning models to discern genuine emotional cues from various modalities. Nonetheless, IEMOCAP is not without its limitations. Despite its rich modalities and emotional labels, the dataset size is comparatively small, potentially limiting its suitability for deep learning models that require larger data volumes. The language constraint to English reduces the model’s versatility across different languages and cultures. The controlled laboratory setting for data collection might also affect the authenticity of emotions, potentially impairing the model’s generalization performance in authentic settings. Furthermore, the dataset manifests a severe class imbalance issue, potentially impacting the model’s accuracy for less represented emotions.

### 2.2. Youtube Dataset

The YouTube dataset was first introduced by Morency et al. in 2011 [25]. The dataset comprises 47 videos, with 20 videos featuring female subjects and the remaining 27 highlighting male perspectives. The dataset further extends its diversity with an age range spanning from 14 to 60, depicting emotive expressions across various stages of life. Although the participants originate from a variety of cultural backgrounds, each video within the dataset is characterized by participants expressing their views in English. This complexity is crucial to the development of models that can robustly function in real-world scenarios where noise is omnipresent. The emotional labeling of the videos in the dataset further augments its value. Each of the 47 videos has been tagged with either positive, negative, or neutral emotion tags. These categorical annotations provide a solid foundation for supervised learning approaches in deep learning. The YouTube dataset contains diverse scenes and environmental noise, which makes it advantageous for improving model generalization. Note that due to the early collection time of the data, the impact of new cultural, social, and technological developments on emotional expression may not be fully reflected in the dataset. Nonetheless, it has served as a cornerstone in the multimodal sentiment analysis field, paving the way for more comprehensive, dynamic, and contemporaneous datasets.

### 2.3. MOUD

The Multimodal Opinion Utterances Dataset (MOUD) was pioneered by Perez-Rosas et al. in 2013 [26]. The dataset offers a unique perspective by focusing on opinion-based utterances, which are intrinsically rich in emotional cues. The MOUD consists of 498 utterances, all painstakingly labeled as either positive, negative, or neutral in emotion. An intriguing aspect of MOUD is its source; the dataset comprises videos curated from YouTube, one of the world’s largest platforms for user-generated content. This provides the advantage of real-world, unscripted emotional expressions, a factor that significantly boosts the applicability and robustness of models trained on this data. The dataset features speakers whose ages range between 20 and 60 years, ensuring a good representation of emotional expressions across different age groups. It includes 15 female speakers, further enhancing its gender diversity. All speakers express their opinions in Spanish, focusing on product reviews. The average duration of the speeches in the dataset is approximately 5 s, which is a typical duration for a single utterance. The dataset includes 182 positive, 231 negative, and 85 neutral utterances. While the MOUD has made significant contributions to the field of MER, it is worth noting that it contains a relatively limited number of samples, with just 498 utterances. This may potentially limit the diversity and the breadth of the emotional expressions it can capture. Nevertheless, the dataset has served as a valuable resource for emotion recognition research, particularly in opinion-based expressions.

### 2.4. ICT-MMMO

The Institute for Creative Technologies’ Multi-Modal Movie Opinion (ICT-MMMO) database, established by Wollmer et al. in 2013 [27], marks a significant contribution to the realm of MER datasets. It assembles videos from two disparate platforms, namely YouTube and ExpoTV. Totaling 370 videos, the dataset leverages the wealth of emotionally charged user-generated content that is ubiquitous on platforms like YouTube. ICT-MMMO sets itself apart with its focus on sentiment analysis of genuine user comments. This real-world context is indispensable for constructing resilient deep learning models. The database houses 228 positive, 57 negative, and 23 neutral YouTube videos, in conjunction with 62 negative ExpoTV movie review videos. Each video in the ICT-MMMO database is classified under one of five emotional states: strongly positive, weakly positive, neutral, strongly negative, or weakly negative. This refined sentiment categorization offers an extension beyond the conventional positive–negative–neutral spectrum, furnishing researchers with a more sophisticated understanding of emotional subtleties and intensities. Nonetheless, it is crucial to recognize the constraints of the ICT-MMMO database, such as its comparably small sample size of 370 videos. Furthermore, it exclusively comprises negative samples from ExpoTV movie reviews. These restrictions could possibly confine the array of emotional expressions the dataset can capture, thereby potentially influencing the broad applicability of models trained on these data.

### 2.5. CMU-MOSI

The Multimodal Opinion-level Sentiment Intensity (CMU-MOSI) dataset, introduced by Zadeh et al. in 2016 [28], marks a significant milestone in multimodal sentiment analysis databases due to its unique characteristic of incorporating subjective sentiment and emotional intensity annotations. The dataset includes 93 randomly selected videos from YouTube, involving 89 different speakers—41 females and 48 males. All these videos were recorded in different settings, some of which used high-tech microphones and cameras while others used less professional recording equipment. In addition, the distance between the users and the camera, as well as the background and lighting conditions, were different. The original quality of the videos remains unaltered, preserving their fidelity. This authentic approach guarantees the data accurately reflect the varying audio-visual quality in user-generated content, thereby providing a robust training set for real-world applications. The CMU-MOSI dataset covers a wide spectrum of topics, including movie and book reviews and product evaluations. The videos are segmented into 2199 clips, each rated on an emotional polarity scale, from +3 (signifying strongly positive sentiment) to −3 (signifying strongly negative sentiment). This scale was developed based on the annotations of five separate annotators, with the average of these five scores serving as the definitive emotional polarity for each clip. This methodology effectively distills the sentiments down to two categories, positive and negative, simplifying the complexity of emotions and providing a clear benchmark for training sentiment analysis models. In summary, the CMU-MOSI dataset’s contribution to the field of MER is invaluable, providing a complex, rich, and diverse set of data for the development and testing of robust, real-world applicable models.

### 2.6. NNIME

The NTHU-NTUA Chinese Interactive Emotion Corpus (NNIME) [29] is a groundbreaking initiative aimed at creating an interactive multimodal corpus for Chinese emotional expressions. This accomplishment is the product of a successful collaboration between Tsinghua University and the National Taiwan University of Arts in Taiwan. The dataset leverages dyadic spoken interactions to evoke genuine emotional responses. Distinctly, the database features spontaneous dyadic spoken interactions involving 44 participants. These interactions have been assiduously captured, producing roughly 11 h of continuous and synchronized data across audio, video, and electrocardiogram modalities. The multimodal character of this dataset extends the scope of sentiment analysis by merging physiological indicators with audio–visual data, setting a precedent for more detailed and holistic emotion assessments. One of the salient characteristics of the NNIME dataset is its extensive annotation process. A robust team of 49 annotators meticulously annotated the data, yielding valuable emotional insights. The annotation procedure includes four distinctive perspectives: peer reports, director reports, self-reports, and observer reports. Such a diversified approach ensures a comprehensive view of the emotional domain, offering researchers profound and expansive insights into the affective phenomena being analyzed. Structurally, the NNIME dataset comprises 6701 sentences and includes both discrete and continuous emotion labels. Discrete labels encompass emotions like anger, sadness, joy, surprise, neutrality, and happiness. Conversely, continuous labels entail the scales of valence and arousal. This two-pronged labeling system captures the subtlety of emotional expression, facilitating a more precise exploration of emotional complexities and variations. Overall, the rigorous process of data collection and annotation employed in creating the NNIME dataset renders it a valuable resource for quantifying and investigating various facets of affective phenomena and human communication. This database’s rich, multimodal nature makes it particularly suitable for developing and validating deep learning models for emotion recognition, thereby pushing the frontiers of MER.

### 2.7. CMU-MOSEI

The CMU Multimodal Opinion Sentiment and Emotion Intensity (CMU-MOSEI) dataset [30] is a crucial resource in multimodal sentiment analysis and emotion recognition, distinguished by its scale, which is unmatched in the field. This dataset encompasses 3837 videos collected from over 1000 distinctive YouTube speakers, with a reasonably balanced gender distribution of 57% male and 43% female speakers, highlighting the dataset’s focus on inclusivity. One of CMU-MOSEI’s unique characteristics is its annotations at the utterance level, with 23,259 samples annotated in this manner. The data collection process incorporated face detection techniques to verify a single speaker’s presence in each video, reinforcing the dataset’s monologic characteristics. This characteristic facilitates individual emotional expression study, thus amplifying the reliability of subsequent sentiment analysis and emotion recognition research. The thematic diversity of the CMU-MOSEI dataset is notable, with a spread across 250 different themes. The three predominant themes are comments (16.2%), debates (2.9%), and consultations (1.8%), while the rest of the themes are nearly evenly distributed. Such thematic richness encourages a comprehensive contextual understanding, enabling a more in-depth exploration of sentiment and emotional expressions across varied discussion scenarios. Although CMU-MOSEI is recognized for its substantial scale, diverse contexts, and inherent authenticity and complexity, it is worth acknowledging the dataset’s limitation—the sole use of English. This constraint could potentially reduce the applicability of models trained on this dataset to other languages. Despite this, the CMU-MOSEI dataset remains a vital resource, offering a broad base for developing and validating models for multimodal sentiment analysis and emotion recognition, thereby advancing research frontiers in this area.

### 2.8. OMG

The One-Minute Gradual-Emotion Recognition (OMG) dataset [31], which incorporates videos from a multitude of YouTube channels, provides a comprehensive view of various emotional behaviors within different contexts. The unique selection process of these videos, centered around the term “monologue”, is designed to depict the gradual evolution of emotions. This approach is integral to the dataset, as it facilitates the modeling of both immediate and long-term emotional trajectories. Every video within the OMG dataset is segmented based on speech, and each of these segments is annotated by at least five independent individuals. This multi-annotator methodology enhances the reliability of the annotations by minimizing individual bias and subjectivity. As part of their assignment, annotators watch the video segments continuously and annotate each one according to the valence/arousal scale and Ekman’s theory of universal emotions. The annotation strategy incorporates both discrete (anger, disgust, fear, happiness, sadness, surprise, and neutral) and continuous (valence and arousal) measures. This combination allows for a holistic examination of emotional states and fosters in-depth investigation and analysis. Comprising 7371 annotated monologue-based speeches, the OMG dataset serves as a robust platform for researchers exploring the dynamism of emotional behaviors within singular narratives. However, the construction of the dataset necessitates certain considerations. The exhibited emotions, influenced primarily by the performers’ interpretive choices, may not genuinely reflect natural emotional expressions. Moreover, the dataset’s reliance on YouTube videos may induce cultural bias skewed towards particular demographics. Despite these potential constraints, the OMG dataset is a formidable resource for the progression of deep learning-based models in multimodal sentiment analysis and emotion recognition. Its focus on gradual emotional change, alongside its detailed annotations, offers researchers an unparalleled opportunity to investigate the complex dynamics of emotional evolution, furthering the boundaries of emotion recognition research.

### 2.9. MELD

In the domain of MER, the Multimodal EmotionLines Dataset (MELD) [32] offers a unique perspective by focusing on the emotional intricacies present in multi-participant dialogue. The dataset is an organized collection of dialogues from the popular American television series “Friends”, featuring 1433 conversations that account for a total of 13,708 utterances. Each utterance within the MELD dataset is annotated with one of seven emotional labels: anger, disgust, sadness, joy, neutrality, surprise, or fear, providing a diverse emotional spectrum for analysis. In addition to these discrete emotion labels, each utterance also includes an emotional classification of positive, negative, or neutral, thereby facilitating a broader understanding of the sentiment behind each statement. The dataset’s design aligns with its primary objective: to furnish training data that are conducive for developing and refining contextual models in dialogue-based emotion recognition. This dataset, therefore, serves as a remarkable tool for investigating emotional dynamics within multi-party dialogue scenarios, a crucial aspect of human communication that remains underexplored in existing datasets. However, the dataset’s realism and cultural applicability must be considered when utilizing it for research or model development. Since the dialogues originate from a scripted television series, they may not accurately reflect the nuances of spontaneous, real-life conversations, thereby limiting the dataset’s authenticity. Moreover, given the American origin of the television series, the dialogues predominantly represent American cultural norms and idiomatic expressions. Consequently, models trained on this dataset may exhibit limitations when deployed in other cultural contexts, highlighting the importance of cultural diversity in emotion recognition research. Despite these limitations, the MELD dataset undeniably provides an enriching foundation for the study of emotion recognition within conversational contexts. It effectively bridges a gap in the literature by focusing on multi-participant dialogues, and can thus guide the development of more sophisticated and context-aware emotion recognition models in the future.

### 2.10. SEWA

The SEWA dataset [33], a significant contribution to the field of MER, comprises a diverse array of audio–visual data gathered using web cameras and microphones. It features over 2000 min of footage capturing the emotional responses of 398 individuals across six distinct cultures, thus providing a comprehensive perspective on multicultural emotional expression. The dataset boasts a gender-balanced pool of participants, with 197 females contributing. The participants span a wide age range from 18 to 65 years, offering valuable insights into the variations in emotional expression and recognition across different age groups. They were recorded while either viewing commercials or discussing them in video chats, settings designed to elicit natural emotional reactions and conversations in response to a variety of stimuli. Such diverse emotional contexts enrich the dataset’s content considerably. The SEWA dataset includes 2562 utterances, each annotated with continuous emotion metrics, i.e., valence and arousal. The multicultural and multilingual nature of the dataset represents one of its primary strengths. By incorporating participants from diverse nations and cultural backgrounds, the dataset broadens its applicability, extending its use to a global scale in the realm of emotion recognition research. However, this cultural diversity poses certain challenges. Variations in emotional expression and interpretation due to cultural differences may result in inconsistencies in the emotion annotations. Such discrepancies could potentially cast doubt on the reliability and consistency of these annotations. These variations underscore the complexity of interpreting emotional behavior across cultures, emphasizing the need for culturally aware emotion recognition systems. Despite these challenges, the SEWA dataset provides a unique opportunity for exploring cultural differences in emotional expressions, as well as for training and validating culturally diverse emotion recognition models.

### 2.11. CH-SIMS

The Chinese single- and multimodal sentiment analysis (CH-SIMS) dataset [34], developed by Yu et al. in 2020, stands as a distinctive contribution to the realm of MER. Consisting of 2281 meticulously refined “in-the-wild” video clips, the average length of each utterance is approximately 3.67 s. In each video clip, only the speaker’s face is visible, thereby eliminating potential distractions or influences from other facial expressions. This unique aspect of the dataset ensures that the emotional state attributed to each utterance pertains exclusively to the speaker, bolstering the accuracy of emotion recognition. The CH-SIMS dataset provides four separate annotations for each utterance: a multimodal annotation and three unimodal annotations. These layered annotations facilitate versatile research avenues, empowering researchers to examine the interplay between different modalities or to conduct unimodal emotion recognition using the distinct unimodal annotations. Emotion categorization within the CH-SIMS dataset is streamlined into five distinct categories: negative, weakly negative, neutral, weakly positive, and positive. This grading system provides a nuanced spectrum of emotional states, further enriching the dataset’s potential for complex emotion recognition tasks. However, despite the advantages offered by the CH-SIMS dataset, certain limitations must be acknowledged. Primarily, the amount of video and audio data compiled within the dataset is relatively limited. Additionally, the dataset exclusively contains Chinese language data, which may inadvertently introduce cultural biases or limitations. While this focus provides an in-depth exploration of Chinese cultural and linguistic emotional expression, it does potentially limit the dataset’s applicability across broader, multicultural emotion recognition research.

### 2.12. CH-SIMS V2.0

In this study, Liu et al. [35] constructed the CH-SIMS v2.0 dataset, which is an enhanced and extended version of the original CH-SIMS dataset. The dataset now includes 4402 supervised data samples, each annotated with unimodal annotations, along with an impressive assortment of over 10,000 unsupervised data samples. A distinctive characteristic of the CH-SIMS v2.0 dataset lies in its diverse array of contexts for data collection. The inclusion of emotional dramas, interviews, modern television shows, talk shows, video blogs, movies, period dramas, variety shows, and other scenarios has ensured that the dataset mirrors the wide-ranging and dynamic environments in which emotions are expressed in real life. This extensive scope enables the dataset to encapsulate a broad spectrum of emotional expressions across various contexts and scenarios, thereby further enriching its potential for intricate emotion recognition research. The primary objective of the CH-SIMS v2.0 dataset is to emulate realistic human–computer interaction scenarios. This goal reflects a critical application of emotion recognition, as understanding users’ emotions can significantly improve the interaction quality and user experience of embodied conversational agents (ECAs).

In conclusion, although existing datasets have undoubtedly propelled the field of MER, there is a need for more comprehensive and diverse datasets to accommodate an increasingly global and multicultural landscape. Continual updates and expansions of these datasets are crucial to align with evolving cultural, social, and technological trends that shape emotional expression. Moreover, the incorporation of varied modalities and contexts in future datasets is recommended to foster more robust and generalizable MER models. Such developments will lay the groundwork for enhanced human–computer interaction, fostering more empathetic and understanding Artificial Intelligence (AI) systems.

## 3. Feature Extraction

In the diverse landscape of MER, three prominent channels of emotional expression take center stage: speech, face, and text. Each channel, or modality, offers a unique perspective on emotion, often carrying nuanced emotional cues that are integral to comprehensive emotion recognition. However, unlocking these cues requires the extraction of key emotional features—a pivotal task in MER that has been greatly refined and evolved with the advent of deep learning techniques. In this section, we delve into the realm of feature extraction, exploring the nuances of each modality and the potent methodologies developed to harness their full potential. Our discussion spans the intricacies of textual, facial, and auditory emotional expressions, spotlighting popular and effective methods that have surfaced since the technology’s widespread proliferation.

### 3.1. Speech Feature Extraction

The emotion-laden speech presents an opportunity to mine acoustic features indicative of the speaker’s emotional state, with the quality of these features exerting a significant influence on the efficacy of emotion recognition. The careful selection of effective speech features that accurately reflect emotional fluctuations is a pivotal challenge within the field of MER. Predominantly, the speech emotion features used can be categorized into two key types: hand-crafted features and deep speech emotion features. The latter are derived using cutting-edge deep learning technologies. This discourse will delve deeper into these categories, shedding light on their unique merits, potential limitations, and the creative methodologies that researchers are employing in the pursuit of augmenting the accuracy of emotion recognition.

#### 3.1.1. Hand-Crafted Feature

Hand-crafted features refer to the features used to describe speech signals that are designed by people based on prior knowledge and professional experience. These features provide us with invaluable insights into the emotional nuances encapsulated within speech, assisting in the complex task of emotion recognition. They are traditionally organized into three main categories: prosodic features, voice quality features, and spectral features.

(1) *Prosodic features*: Emotions precipitate physiological changes that directly impact speech production and, thus, significantly affect speech intonation [36]. Prosodic features, capturing variations in pitch, tone, rhythm, and stress in speech, serve as robust indicators of these emotional shifts. As a result, these features have become indispensable in the field of emotion recognition and are widely acknowledged by researchers [37,38]. The array of prosodic features contributing to the depiction of emotions in speech encompasses, among others, fundamental frequency (F0), energy, and speech rate [39,40,41,42]. Among them, F0 is considered one of the most crucial features to predict emotional states in speech. Relevant studies have shown that people’s F0 tends to be higher with a wider range when they are in an excited emotional state such as anger, lower and with a narrower range when they are in a depressed emotional state such as sadness, and relatively stable when they are in a calm emotional state [42]. In addition to F0, energy (i.e., intensity) in speech is also a vital feature. Scherer et al. [38] found that high-frequency energy increases when people experience fear, happiness, and anger, while high-frequency energy decreases during times of sadness. Furthermore, Nwe et al. [43] pointed out that emotions with higher activation levels, such as anger, surprise, and happiness, generally have higher intensity, while emotions like fear, sadness, and disgust have lower intensity. Furthermore, speech rate, the speed at which words are spoken, greatly affects emotion detection. Research indicates that the speech rate tends to be faster for fear, disgust, anger, and happiness; normal for surprise; and slower for sadness [44]. Consequently, fluctuations in the speech rate serve as a vital cue in distinguishing different emotional states. In conclusion, the careful analysis and extraction of prosodic features offer a comprehensive, although not exhaustive, approach to understanding and recognizing emotional states through speech. They form a crucial part of hand-crafted features used in MER, with each contributing uniquely to the richness of emotion recognition. Nevertheless, it is important to remember that prosodic features form just one aspect of emotional recognition, and should be used in concert with other features for an all-encompassing, nuanced understanding of emotions.
(2) *Voice quality features*: Voice quality features are primarily utilized to assess the purity, clarity, and intelligibility of speech. These are affected by acoustic performances such as wheezing, choking, and tremors, which frequently arise when the speaker is emotionally excited or has difficulty calming down. Voice quality features include formant, harmonic-to-noise ratio (HNR), jitter, and shimmer. The formant reflects the characteristics of the vocal tract, which is a significant parameter impacted by emotional speech. According to previous research findings, compared with neutral speech, the first formant frequency of happy and angry speech is slightly higher, while the first formant frequency of sad speech is significantly lower [36,42]. Another crucial voice quality feature is the HNR, which represents the ratio of harmonic components to noise components in speech. It is worth noting that the noise in this context is not environmental noise, but glottal noise arising from incomplete closure of the vocal folds during speech production. Jitter is a term for the degree of rapid and repetitive changes in the measured fundamental frequency, which mainly indicates roughness in the sound and, to a lesser extent, the degree of hoarseness commonly observed in anxious speech. Shimmer mainly demonstrates the degree of hoarseness by reflecting changes in the amplitude of voice between adjacent vibratory cycles. In conclusion, voice quality features play an indispensable role in extracting valuable insights from speech for emotion recognition. The concurrent use of these features substantially enhances the breadth and depth of emotion recognition, thus facilitating a more sophisticated comprehension of emotional states in speech.
(3) *Spectral features*: Spectrum-based correlation features are usually short-term representations of speech signals, as speech signals are generated from the coordinated movement of multiple articulators, and the physical characteristics of the articulators determine that speech is difficult to change significantly in a short period of time. Typically, speech signals ranging from 5–50 ms are relatively stable. When recognizing emotions in speech, the most important perceptual properties are reflected in the power spectrum. Emotional expression in speech has a great impact on the distribution of spectral energy in different frequency bands. Spectral features encompass linear and cepstral features, wherein the former involves Linear Prediction Coefficients (LPC), and Log-Frequency Power Coefficients (LFPC), and the latter involves Mel Frequency Cepstral Coefficients (MFCC) and Linear Prediction Cepstral Coefficients (LPCC). Among them, MFCCs, arguably the most commonly used feature in speech signal processing, capture the power spectrum of speech on a nonlinear mel scale, which approximates the human auditory system’s response. The use of the mel scale makes MFCCs particularly valuable when capturing perceptually relevant spectral shapes, thereby enabling effective emotion recognition. To conclude, spectral feature extraction and analysis present a robust methodology for comprehending and recognizing emotional states via speech. Each feature type provides unique insights, collectively contributing significantly to the richness of emotion recognition in multimodal emotion recognition frameworks. However, it is pivotal to remember that spectral features represent merely one facet of emotion recognition and should be employed in combination with other features for a comprehensive and nuanced emotional understanding.

In current MER, it is common practice to use OpenSMILE [45], COVAREP [46], OpenEAR [47], and librosa [48] to extract the aforementioned acoustic features that represent the emotional characteristics of the speech modality. Furthermore, the robustness and generalizability of these features may vary across different speech contexts and individual speakers, owing to the subjective and dynamic nature of emotional expression. Therefore, while hand-crafted features provide us with a powerful tool to harness emotional cues from speech, they should be used in conjunction with other methodologies and modalities to attain a comprehensive understanding of emotional states. This serves to enhance the richness and precision of emotion recognition in a multimodal setting.

#### 3.1.2. Deep Feature

Although hand-crafted features can describe the basic acoustic properties and emotional information of speech signals well, this extraction method requires prior knowledge and professional experience, and it is difficult to cover all speech emotion features. As a result, an increasing number of researchers are turning to deep learning technology to extract speech features automatically in the current field of SER. These methods typically involve using deep learning models to learn how to represent speech signals and generate a set of feature vectors that represent their deep features. Currently, a common approach involves using self-supervised training methods to learn high-quality representations from unlabeled data, and then using these representations to replace hand-crafted features to complete downstream tasks. This is a research hotspot in the field of speech emotion [49,50,51,52,53]. For example, Schneider et al. [54] proposed an unsupervised speech pre-training method named wav2vec that only uses unlabeled speech data for self-supervised training to obtain generalized speech features that can be applied to multiple tasks. Note that wav2vec directly trains on speech’s raw waveform to extract deep features that represent speech signals. Subsequently, Baevski et al. [55] developed a new self-supervised learning framework for speech named wav2vec 2.0. Empirical evidence underscored the superior performance and generalization of wav2vec 2.0 over its predecessor. Hsu et al. [56] introduced a novel self-supervised learning framework for speech representation learning named HuBERT, which employs a more direct predictive loss by separating the acoustic unit discovery step from the masked prediction representation learning phase than wav2vec 2.0. Most recently, Chen et al. introduced WavLM [57], the first universal speech pre-training model capable of being applied to a broad range of tasks, including speaker recognition, speech recognition, and emotion recognition. Experimental results indicate that WavLM outperforms all preceding models on these tasks, pointing to the promise and potential of deep learning in enhancing SER. Despite these advancements, there are challenges inherent to deep feature extraction in SER. Deep learning models require large volumes of data for training, which may not always be available or feasible to obtain in specific contexts. Additionally, the self-supervised learning models, though efficient, still struggle with differentiating finer emotional nuances in speech. Finally, despite the high performance of models like WavLM, they are computationally intensive and demand substantial resources, posing a challenge for real-time applications. Future research must aim to address these challenges to further enhance the effectiveness and applicability of deep feature extraction in SER.

### 3.2. Textual Feature Extraction

Textual emotions are typically conveyed through textual information, and extracting emotional features from text is critical to recognizing emotions in text. Traditional methods for extracting emotional features use the bag-of-words (BoW) model [58,59]. Although the BoW model has straightforward processing steps that are easy to understand, it neglects word order and grammar relationships, resulting in a loss of contextual information. As a consequence, it is unable to capture the connections and structured information between words. Word embedding techniques have been proposed to overcome these limitations. Word embedding is a feature extraction technique that uses neural networks to learn representations of text, such that words with similar meanings have similar representations. Pre-trained deep learning-based word embedding models have been widely applied in text emotion recognition tasks. Earlier word embedding models, e.g., word2vec [60] and GloVe [61], predominantly relied on syntactic contexts for training. These models were trained on vast amounts of unlabeled data, aiming to capture intricate grammatical and semantic rules. Pre-trained word embedding models generally perform better than those initialized randomly. However, a limitation of these early models is their assumption that every word has a unique vector representation, irrespective of its monosemous or polysemous nature. This limitation overlooks diverse contextual information, compromising the models’ effectiveness.

To mitigate the highlighted limitations, Peters et al. introduced the ELMo (Embeddings from Language Models) [62] method. It produces deep contextualized word embeddings by capturing semantic variations based on context. Distinct from conventional language models, ELMo adopts a profound bidirectional architecture, accounting for both left and right contexts within a sentence. This structure can capture intricate word features, such as synonyms and polysemy, across different linguistic contexts. Later, Devlin et al. introduced BERT (Bidirectional Encoder Representations from Transformers) [63], a transformer-based encoder with bidirectional contextualized word representations. Unlike traditional language models, BERT simultaneously considers both left and right contexts to predict the next word in a sentence. It pre-trains deep, bidirectional contextualized word representations from large amounts of unmarked text through unsupervised learning.

Nonetheless, it is imperative to note that the models mentioned above do not explicitly account for emotional information. Addressing this gap, Tang et al. [64] pioneered the Sentiment-Specific Word Embeddings (SSWE). Empirical results have shown its superior efficacy over other embeddings in integrating emotion labels at the word level for emotion-centric tasks. Following this, Xu et al. [65] unveiled Emo2Vec, embedding emotional semantics into fixed-size vector representations at the word level. Experiments revealed that Emo2Vec surpasses conventional word embedding techniques in numerous emotion-driven tasks. Moreover, Emo2Vec’s training methodology bears significant relevance for feature extraction in diverse domains.

### 3.3. Facial Feature Extraction

Facial expressions have always been essential to recognizing the emotions of the speakers in MER. Therefore, obtaining features that represent facial emotional information for affective discrimination is imperative. Traditional facial feature extraction methods include Local Binary Pattern (LBP) [66], Active Appearance Model (AAM) [67], Active Shape Model (ASM) [68], Scale Invariant Feature Transform (SIFT) [69], Histograms of Oriented Gradient (HOG) [70], and Gabor Wavelet Transform [71]. However, traditional facial feature extraction methods require manual feature extraction, which is time-consuming and labor-intensive. Because facial semantic information is more diverse than that of other images, manual feature extraction can overlook significant semantic information. Deep learning models, especially convolutional neural networks (CNN), can automatically learn the optimal features without prior intervention, eliminating the tedious process of manual feature extraction. Furthermore, deep learning frameworks can perform robust and accurate feature learning in supervised and unsupervised settings, and generate optimal performance in various applications, including digital recognition, image classification, and feature learning. Consequently, an increasing number of researchers have adopted deep learning algorithms to extract facial features in affective computing tasks. For instance, Tran et al. [72] proposed a simple and efficient three-dimensional convolutional neural network (3D-CNN) for extracting spatiotemporal features. This model can be used for multiple tasks, such as action recognition and dynamic scene recognition. Yu et al. [73] proposed a new architecture called spatio-temporal convolutional features with nested LSTM (STC-LSTM), which is based on three deep learning subnetworks: 3DCNN, T-LSTM, and C-LSTM. 3DCNN is used for extracting spatiotemporal features, T-LSTM is used for maintaining temporal dynamics, and C-LSTM is used for modeling multi-level features. At present, commonly utilized libraries consist of OKAO, Computer Expression Recognition Toolbox (CERT) [74], OpenFace [75], and FACET. OKAO Vision detects and extracts facial features from individual frames, providing measures of smile intensity (ranging from 0 to 100) and eye gaze direction. The CERT can automatically extract smiles and estimates head poses and facial action units. The OpenFace 2.0 toolkit provides data on 68 facial landmarks, 17 facial action units, head poses, head orientation, and eye gaze. Finally, the FACET library extracts a multitude of visual features including facial action units, facial landmarks, head poses, gaze tracking, and HOG features [76].

## 4. Multimodal Fusion Methods

**Figure 2:**
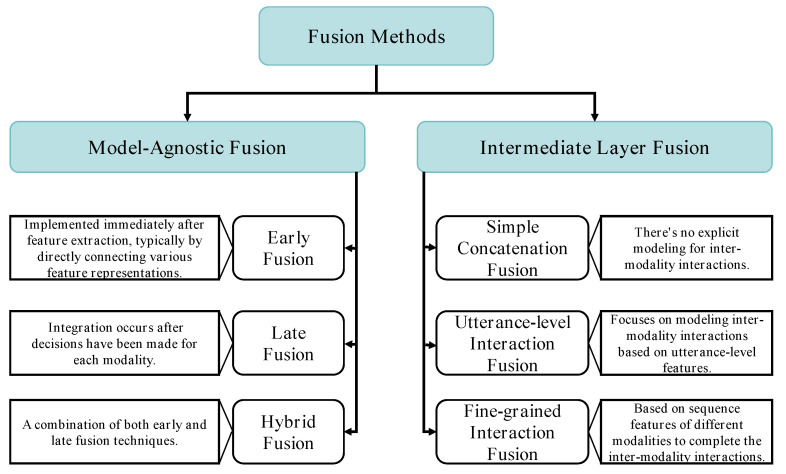
Multimodal fusion methods for multimodal emotion recognition.

Emotion, as a complex interplay of psychological and physiological processes, operates largely via non-linguistic means, thereby adding a layer of complexity to its recognition. Unimodal information, limited in expressiveness by nature, often falls short of capturing the full spectrum of emotional states. This limitation emphasizes the need for the integration of multiple modalities of information, leading to a more comprehensive understanding of emotions and subsequently improving emotion recognition efficacy. Various experimental studies have attested to the superior performance of MER models over unimodal ones, placing MER at the vanguard of research within affective computing [77,78,79,80]. The crux of MER lies in its fusion process, which amalgamates varied types of signals to derive complementary information, which is mutually supportive across the modalities. This crucial step significantly contributes to improving the overall performance of the MER model. The fusion of different types of signals to obtain mutually supportive complementary information is central to improving model performance in MER. We categorize the current deep learning-based MER fusion methods into two main types. The first type is model-agnostic fusion methods, where the fusion process is not directly dependent on a specific deep learning model. The second type is fusion methods based on the intermediate layers of the deep models (i.e., intermediate layer fusion), which performs the fusion within the deep network. For an overview of these methods, please refer to Figure 2.

### 4.1. Model-Agnostic Fusion

Model-agnostic fusion methods are sub-categorized as early fusion, which integrates immediately after feature extraction, usually by simply connecting various feature representations; late fusion, which consolidates decisions (like classification or regression) subsequent to individual modality decision-making; and hybrid fusion, an amalgamation of both early and late fusion techniques. Although model-agnostic fusion methods can be implemented using nearly any single-modality classifier or regressor, they are not typically crafted for multi-modal data [81]. In the subsequent sections, we will delve deeper into these fusion techniques.

#### 4.1.1. Early Fusion

**Figure 3:**
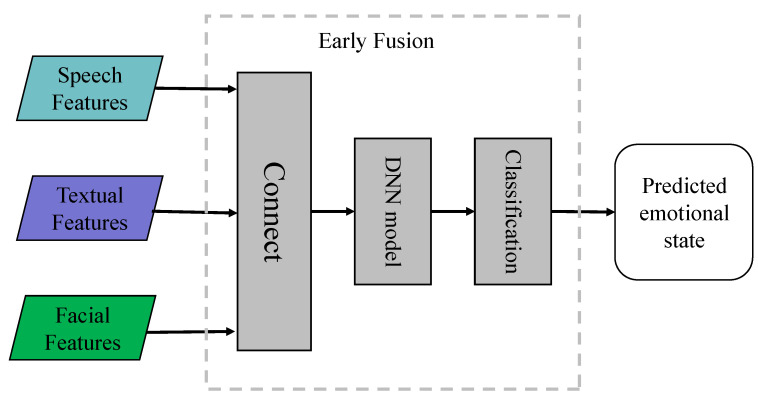
Multimodal emotion recognition framework based on early fusions.

In the realm of MER, early fusion, synonymous with feature-level fusion, holds significant prominence. Figure 3 shows the framework of multimodal emotion recognition based on early fusion. It is characterized by its simplicity and lower computational complexity. In this approach, features from various modalities, e.g., text, speech, and vision, are combined through a concatenation operation. These amalgamated features are then utilized as inputs for a Deep Neural Network (DNN) to undergo classification training. Poria et al. [82] were pioneers in the implementation of this fusion method. They extracted feature vectors from the speech, vision, and text modalities, amalgamated them into a comprehensive feature vector, and then utilized this unified vector as an input for their classification model. This laid the groundwork for further explorations in early fusion. In a following study, Huang et al. [83] enhanced data richness to evaluate the efficacy of early fusion. This was achieved during the 2018 Audio/Visual Emotion Challenge (AVEC), in which the researchers augmented the training data by replacing the original training samples with shorter overlapping samples extracted from them, thus multiplying the number of training samples. This amplification of training data diversity, coupled with the implementation of early fusion, illuminated the potential of this method. Further progress in early fusion was seen in the work of Williams et al. [84].They constructed a multimodal fusion system that discerns emotions and their intensities. By carrying out feature fusion at the input level and employing a Bidirectional Long Short-Term Memory (BLSTM) model for sequence learning, they underscored that early fusion can effectively predict the presence of multi-label emotions and their coarse-grained intensities. The primary advantage of early fusion is its ability to enable early correlations among distinct multimodal features, thereby significantly improving the model’s emotion recognition performance. This advantage has solidified the role of early fusion in MER applications. Nevertheless, the simplicity of early fusion also presents certain limitations. The mere concatenation of different modal data might result in the model’s inability to process the unique features of each modality independently. As a result, vital emotional information from certain modalities may be overlooked. Additionally, this straightforward concatenation method cannot effectively filter out conflicting or redundant information from different modalities. Additionally, early fusion faces a critical challenge in addressing the time synchronization issue across various data sources. Given that features harvested from different modalities have varying time scales, measurement levels, and temporal structures, achieving harmony among them poses a complex task. In summary, while early fusion has proven instrumental in MER, researchers must address its limitations to optimize this method further. This ongoing exploration offers a fascinating area of focus in the continuing evolution of MER.

#### 4.1.2. Late Fusion

**Figure 4:**
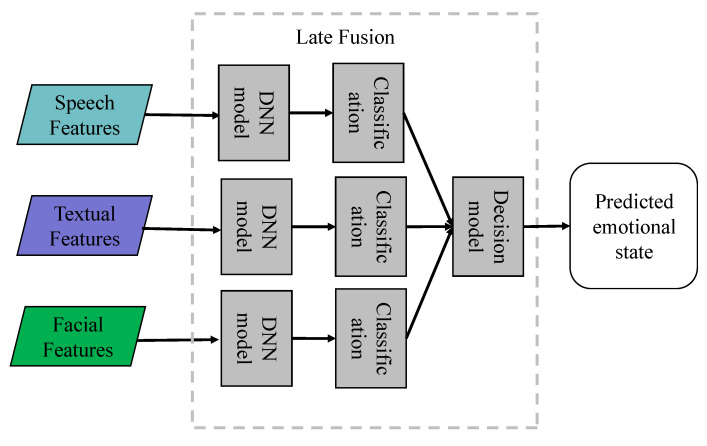
Multimodal emotion recognition framework based on late fusions.

Late fusion methods, also referred to as decision-level fusion methods, are widely employed in multimodal affect recognition. The basic idea is to independently extract features and train models for each modality, and then combine their prediction results through averaging, weighted sum, majority voting, or deep neural networks to obtain the final emotion category. We show late fusion in Figure 4. Due to its feature-independent fusion process and uncorrelated mistakes from different classifiers, this fusion method has garnered significant attention. Several researchers have demonstrated the application of this method. Poria et al. [85], for example, developed separate unimodal affect recognition models for speech, text, and visual modalities in 2016. Following the training phase, these models produced prediction probabilities for each modality, which were then integrated through weighted fusion to produce the final prediction. Furthermore, Huang et al. [86] adopted Long Short-Term Memory (LSTM) networks to create affect prediction models that fully leverage affective information from each modality. They formulated prediction models for various feature types within each modality and used Support Vector Regression (SVR) as the fusion model in the decision layer. In an attempt to augment performance, Su et al. [87] proposed a multi-level segmented decision-level fusion model for affect recognition. Their model incorporated Bidirectional LSTM (BLSTM) as a multi-level segmental affect feature learning model and SVR as the fusion model at the decision level. Notably, the BLSTM was capable of effectively modeling different forms of affective information, considering the influence of previous and subsequent affective features on the current outcome. Meanwhile, SVR assisted in compensating for redundant information in affect recognition. Recently, Sun et al. [88,89] employed LSTM and a self-attention mechanism to capture complex temporal dependencies in feature sequences. They then fused the prediction results of several unimodal affect recognition models using a second-level LSTM as the fusion model. Compared to feature-level fusion, decision-level fusion is simpler, more flexible, and does not require the temporal synchronization of modality features. Additionally, during the late fusion process, each modality can utilize the most suitable classifier or model to learn its features, thus leading to improved local decision outcomes. However, late fusion presumes that each modality operates independently, thereby ignoring the interaction information between different modalities and potentially limiting the accuracy of the final affect prediction. Future investigations should explore fusion methods that preserve the advantages of late fusion while incorporating inter-modality interactions to enhance affect recognition accuracy.

#### 4.1.3. Hybrid Fusion

**Figure 5:**
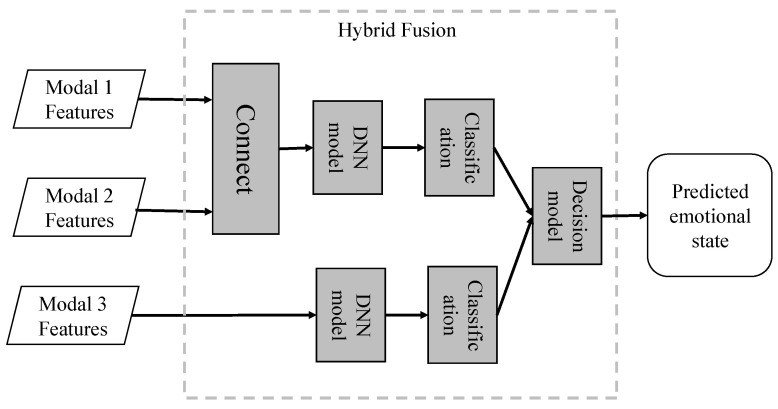
Multimodal emotion recognition framework based on hybrid fusions.

Unlike the previously discussed early, late, and intermediate fusion methods, hybrid multimodal fusion provides a distinct approach, integrating the strengths of both early and late fusion strategies, as depicted in Figure 5. The goal of this fusion method is to maximize the utilization of emotional information extracted from multimodal data, while also considering the interplay and synergy between different modalities. For example, Wollmer et al. [27] proposed a hybrid fusion method that uses Bidirectional Long Short-Term Memory (BLSTM) to merge audio and visual features at the feature level. Following this, they integrated the fused outcomes with the predictions from a text classifier using decision-level fusion, a strategy typically found in late fusion methods. By using this hybrid approach, their method benefits from both the high-level abstraction of feature interactions provided by early fusion and the modality-specific expertise of separate classifiers provided by late fusion. Within the field of Music Emotion Recognition (MER), Nemati et al. [90] introduced a comparable hybrid fusion methodology. Initially, they mapped the speech and visual modalities onto a shared latent space using feature-level fusion, a technique similar to early fusion methodologies. These resultant latent features are subsequently utilized for emotion classification. Concurrently, they train an independent supervised classification model using text features. In the final stage, they apply a decision-level fusion method, underpinned by the Dempster–Shafer theory, to amalgamate the outcomes of the text and audio–visual classifications. This hybrid strategy enables the utilization of inter-modality interactions at an early phase while preserving the flexibility of independent modality classification at a later stage. To conclude, hybrid multimodal fusion techniques endeavor to exploit the unique advantages of both early and late fusion methods, aiming to deliver a more resilient and comprehensive affect recognition system. Nonetheless, the complexity of these methods also presents challenges, particularly when it comes to determining the optimal blend of early and late fusion strategies and managing the escalated computational demands. Consequently, ongoing research and development in hybrid fusion methods remain a promising and critical direction for the advancement of the field of multimodal affect recognition.

### 4.2. Intermediate Layer Fusion

Diverging from the previously mentioned early, late, and hybrid fusion techniques, intermediate fusion designates a distinctive approach that focuses on the fusion of modalities within the network layer of deep neural network models. This technique bestows upon multimodal recognition models the capacity for comprehensive, end-to-end emotion recognition, while fully modeling the intra- and inter-modality interactions. The strength of intermediate fusion lies in its ability to capitalize on the strengths of deep learning, namely its capacity to learn intricate patterns and represent high-dimensional data effectively. This stands in contrast with early fusion methods, which may limit the capability to model complex interactions due to preprocessing steps, and late fusion techniques, which often lack the capacity to capture detailed inter-modality relationships due to the segregation of modality training. Notably, the application of intermediate fusion is not a one-size-fits-all method. The focus of fusion methods, based on deep model intermediate layers, is the modeling of interactions between modalities. These methods can be further categorized based on the type of interaction between modalities as follows: simple concatenated fusion, which does not explicitly model inter-modality interactions; utterance-level interaction fusion, which models inter-modality interactions based on utterance-level features; and fine-grained interaction fusion, which is based on sequence features of different modalities to complete the inter-modality interactions.

#### 4.2.1. Simple Concatenation Fusion

Simple concatenation fusion based on deep neural networks refers to the process in which MER models independently analyze the input features of different modalities and directly concatenate these features as the input of the next layer network to learn the interaction information between modalities. Nguyen et al. [91] proposed a novel multimodal sentiment recognition model that utilizes three convolutional neural networks to separately process the low-level features of speech, text, and visual modalities. The aim is to attain high-level emotional feature representations. Subsequently, the feature vectors from each modality are merged into a single vector and injected into the emotion classification network for sentiment evaluation. Similarly, Tripathi et al. [92] adopt the optimal feature extraction network for each modality and execute concatenation fusion in the final layer for classification. Experimental evidence has proven that this approach can create more robust and precise emotion detection models. Ortega et al. [93] proposed a new deep neural network for multimodal fusion emotion recognition in audio, video, and text modalities. This deep neural network architecture includes independent and shared layers, aiming to learn the representations of each modality as well as the optimal combined representation to achieve the best prediction performance. Experimental results achieved using the AVEC wild emotion analysis dataset indicate that the proposed deep neural network model outperforms early and late fusion approaches. Liang et al. [94] postulate that despite the potential misalignment of emotional expressions at the frame level across different modalities, the overall emotional states should exhibit similarity at a coarse-grained discourse level. Leveraging this assumption, they train the encoder via semi-supervised learning, using unlabeled video data to secure improved encoder representations. Given that the representation vectors obtained for each modality are similar, the authors simply concatenate them and conduct direct classification. Yu et al. [95] proposed a self-supervised multi-task multimodal sentiment analysis network (Self-MM). In the Self-MM model, representations from different modalities are fused using the simplest concatenation method. Despite the apparent similarity between simple concatenation fusion and early fusion, noteworthy distinctions exist between the two. Furthermore, Han et al. [96] pointed out that the aforementioned methods lack control in the information flow from raw input to fused embedding, which may lead to information loss and an increased risk of unintended noise. To address this issue, they introduced the concept from information theory—mutual information (MI)—and proposed a hierarchical mutual information maximization framework for MER (i.e., MultiModal InfoMax, MMIM). In MMIM, MI maximization is performed at both the input and fusion layers. By enhancing the MI between multi-modal inputs, it can effectively filter out modality-specific noise irrelevant to the task while preserving modality-invariant content across all modalities. MMIM also applies MI maximization between the fusion result and input modalities again, ensuring that the fusion output sufficiently captures modality-invariant clues among the modalities. Like Han et al. [96], Zheng et al. [97] also introduced a multimodal representation model, MMMIE, grounded in the principles of mutual information maximization, minimization, and identity embedding. This model aims to maximize the mutual information between modalities while minimizing the mutual information between input data and its features, extracting modality-invariant and task-related information. Moreover, by incorporating identity embedding, MMMIE bolsters the context-aware capabilities of the downstream network. Subsequently, Mai et al. [98] aimed to minimize redundancy in the fusion process by introducing the Multimodal Information Bottleneck (MIB) for MER. This method provides a non-redundant, robust multimodal representation for MER. Drawing inspiration from the general Information Bottleneck (IB) principle, the MIB is designed to learn the minimal sufficient representation for a given task. It accomplishes this by maximizing the mutual information between the representation and the target while simultaneously constraining the mutual information between the representation and the input data. In simple fusion, the model conducts modality-specific modeling on each modality’s features prior to executing feature concatenation fusion. Therefore, simple concatenation fusion not only addresses the issue of modality-specific modeling, which cannot be attained via early fusion, but also eradicates the need to consider the temporal synchronization of different modalities’ features. However, simple concatenation fusion might not fully learn the interaction information between modalities, posing a limitation. This overview of simple concatenation fusion underscores its utility in MER while also acknowledging its potential limitations. As research in this field advances, the exploration of other fusion methods and their combination with simple concatenation fusion may hold promising potential for further enhancing emotion recognition systems.

#### 4.2.2. Utterance-Level Interaction Fusion

The technique of utterance-level interaction fusion explicitly models the features at the utterance-level across different modalities instead of merely concatenating them. Importantly, utterance-level features represent the emotional information of each modality by utilizing a single feature vector. One of the pioneers in this area is Zadeh et al. [99], who proposed the Tensor Fusion Network (TFN) for MER. The TFN consists of three primary components: modality embedding subnetworks, a tensor fusion layer, and a sentiment inference subnetwork. In the first stage, modality embedding subnetworks model intra-modality dynamics, extracting a rich modality embedding, namely, the utterance-level features. Next, the tensor fusion layer models unimodal, bimodal, and trimodal interactions using a three-fold Cartesian product derived from utterance-level features. Lastly, the sentiment inference subnetwork conducts sentiment inference. Continuing the exploration in this field, Liu et al. [100] proposed Low-rank Multimodal Fusion (LMF) to achieve the interaction between modalities based on utterance-level features. The experiment confirmed that the LMF effectively improves the training and testing efficiency compared to TFN. Following the inception of TFN and LMF, various other methodologies were proposed to further improve TFN. These include Temporal Tensor Fusion Network (T2FN) [101], Hierarchical Feature Fusion Network (HFFN) [102], Modality-based Redundancy Reduction Fusion (MRRF) [103], and Semi-Tensor Product (STP) [104]. Each of these methodologies contributes unique perspectives and solutions to MER. Notably, Mai et al. [105] introduced a pioneering adversarial encoder–decoder classifier framework aimed at acquiring a modality-invariant embedding space. Considering the distinct distributions across modalities, the authors employed adversarial training to align the distribution of the source modality with that of the target modality. Moreover, the embedding space was subject to additional constraints by incorporating reconstruction loss and classification loss. Lastly, a hierarchical graph neural network was employed to integrate the encoded representations, explicitly exploring the interactions between unimodal, bimodal, and trimodal information across multiple stages. Additionally, Wu et al. [106] developed a fusion network named Bimodal Information-augmented Multi-Head Attention (BIMHA), which relies on multi-head attention and comprises a total of four layers. The first layer captures modality-specific dynamics within a single view. The second layer represents cross-view dynamics. In the third layer, bimodal interaction information is extracted by utilizing a multi-head attention mechanism, which calculates bimodal attention for weight allocation in feature attention. Lastly, the independent modality embeddings are concatenated with bimodal attention to form a multimodal representation used for predicting the emotion of each utterance. Recently, Liu et al. [107] devised a multimodal fusion network with complementarity and importance for emotion recognition. This network takes into account the varying importance of different modalities and employs an importance attention network to assign weights accordingly. Additionally, given the complementary relationship between modalities, this approach constructs a complementary attention network to enhance feature fusion and achieve effective interaction among multimodal features. In summary, utterance-level interaction fusion has proven to be an effective approach for MER, with significant strides made in the development of various network structures and fusion methods. As the field continues to progress, further exploration and refinement of these methodologies is expected to improve both the efficiency and accuracy of MER.

#### 4.2.3. Fine-Grained Interaction Fusion

Although multimodal feature fusion methods based on utterance-level features can use global features for prediction, they ignore fine-grained interactions between modalities in the modeling process, which may fail to capture local information [108]. Hence, another option is multimodal feature interaction methods based on fine-grained features. Multimodal streams collected from different modal sequences often exhibit inherent asynchrony due to variations in their receiving frequencies. Since the fine-grained inter-modality interaction needs to match the alignment relationship between modalities, the fine-grained features are often divided into aligned word-level features and unaligned features. Aligned word-level features refer to features that have been forced to align before interacting. To extract word-level features, the first step is to apply forced alignment to obtain timestamps for each word, including start and end times. The utterance is then divided into segments according to the timestamps. Finally, word-level acoustic or visual features are obtained by averaging frame-level features of speech segments. Based on word-level features, many methods for word-level multimodal MSA have been proposed. To the best of our knowledge, Chen et al. [109] first based the idea of word-level modality fusion to propose a gated multimodal embedding LSTM with temporal attention (GME-LSTM(A)) model, which consists of two modules, i.e., gated multimodal embedding and LSTM with temporal attention. The gated multimodal embedding alleviated the difficulty of fusion when noisy modalities were present. The LSTMs with temporal attention performed word-level fusion between input modalities at a finer interaction and focused on the most significant time steps. Zadeh et al. [110] proposed a novel network for understanding human communication based on word-level features called Multi-Attention Recurrent Network (MARN). MARN includes two key components: long-short-term mixed (LSTHM) memory and multi-attention blocks (MAB). Hybrid memory LSTHM was an extension of LSTM that carries mixed information by reconstructing memory components. MAB was used to discover cross-view dynamics across different modalities. LSTHM was expanded at the word level at time steps, while MAB was applied at each time step to achieve fine-grained interactions between modalities. Subsequently, Zadeh et al. [111] proposed a memory fusion network (MFN) for multi-view sequential learning, which also used word-level features as interactive features. The MFN first used the LSTMs module system to model the intra-modality interaction, then used the delta-memory attention network (DMAN) module to achieve fine-grained inter-modality interactions. Zadeh et al. [30] introduced a novel multimodal fusion technique known as Dynamic Fusion Graph (DFG) to investigate the interplay between modalities in human multimodal language. DFG is an improved version of the MFN in which the original fusion method, DMAN, has been replaced with DFG. The resulting model is named the Graph Memory Fusion Network (Graph-MFN). Furthermore, there are several other enhanced models based on MFN, e.g., M3ER [112]. Liang et al. [113] presented Recurrent Multistage Fusion Network (RMFN), which decomposes the fusion problem into multiple stages. Each stage focuses on a subset of multimodal signals to achieve specialized and effective fusion. This multi-stage fusion approach models cross-modal interactions by leveraging the intermediate representations from previous stages. Integrating this fusion method with a recurrent neural network system enables the simulation of temporal and intra-modal interactions.

According to the above description, the aforementioned studies based on word-level features require forced alignment of text and speech, which is time-consuming and labor-intensive. To handle this problem, researchers have begun to study fine-grained interactions between modalities based on unidentified features. In this case, the core problem is how to match the alignment relationship between modalities automatically. Since the attention mechanism can measure the similarity between features, the feature alignment between modalities can be achieved based on the attention mechanism. Currently, multimodal MER based on attention mechanism has attracted widespread attention. For example, Tsai et al. [114] proposed a multimodal Transformer (MULT) for unaligned multimodal language sequences. The core of the Mult model is the crossmodal attention, which attends to fine-grained interactions between multimodal sequences across distinct time steps and latently adapts streams from one modality to another. Although the multimodal Transformer (MulT) method extends the self-attention mechanism of the original Transformer network to learn cross-modal dependencies between elements, directly copying the self-attention is influenced by the mismatch between different modal features, leading to potentially unreliable cross-modal dependencies. Based on this observation, Liang et al. [115] introduced the Modality-Invariant Cross-Modal Attention (MICA) method, which learns cross-modal interactions in a modality-invariant space and effectively solves the sequence matching problem in unaligned features. The effectiveness of the MICA method has been validated through experiments on multiple datasets. Additionally, to tackle the problem of cross-modal asynchrony in multimodal sequences, Lv et al. [116] introduced the Progressive Modality Reinforcement (PMR) method, which relies on cross-modal Transformers. This method incorporates a message hub that facilitates information exchange among modalities. The message hub sends mutually shared messages to each modality, reinforcing their features through cross-modal attention. Simultaneously, the message hub accumulates reinforced features from each modality and leverages them to generate enhanced common messages. This iterative process enables the gradual complementary integration of shared information and modality-specific features. Ultimately, the enhanced features are employed for sentiment prediction tasks. Tzirakis et al. [117] presented a textual architecture based on Transformers and an attention-based fusion strategy to effectively integrate diverse modal features and enhance sentiment recognition performance. The proposed textual model employs multilinear projection and context-aware feature generators to capture sentence semantics. Moreover, the proposed fusion strategy achieves superior balance among the relationships across different modalities compared to a straightforward concatenation approach, resulting in enhanced recognition performance. In summary, fine-grained interaction fusion methods provide an innovative avenue for capturing nuanced interactions between modalities, proving to be pivotal in advancing MER. These methods ensure that both global and local information is integrated for precise emotion recognition. While advancements have been made in this sphere, continuous research is warranted to refine these methods further and develop more robust systems for MER.

## 5. Evaluation Metrics

When we delve into deep MER models, we find that their methods of handling continuous emotions and discrete emotions differ. For the prediction of discrete emotions, the model typically employs the cross-entropy loss function, which is a commonly used loss function in classification problems, aiming to minimize the difference between the probability distribution predicted by the model and the probability distribution of the true labels.

(1) $$L=-\sum_{i=1}^{N}y_{i}\log\left(p_{i}\right)$$

where *N* is the number of classes, $y_{i}$ is the true label, and $p_{i}$ is the probability predicted by the model for that category.

As for the prediction of continuous emotions, due to their nature leaning more toward regression problems, the model tends to use MSE (Mean Squared Error) and MAE (Mean Absolute Error) as loss functions.

(2) $$\mathrm{MSE}=\frac{1}{M}\sum_{i=1}^{M}{\left(y_{i}-{\hat{y}}_{i}\right)}^{2},\mathrm{MAE}=\frac{1}{M}\sum_{i=1}^{M}\left|y_{i}-{\hat{y}}_{i}\right|$$

where *M* is the total number of samples, $y_{i}$ is the true continuous emotion value, and ${\hat{y}}_{i}$ is the predicted value by the model.

As a result, in this survey, we summarize two primary types of evaluation metrics for emotion recognition models: one for classification models, denoted as classification evaluation metrics, and another for continuous emotion prediction models, referred to as regression evaluation metrics.

### 5.1. Classification Evaluation Metrics

#### 5.1.1. Weighted Average Accuracy (ACC)

The ACC is a widely adopted evaluation metric for emotion classification tasks. It considers the imbalanced distribution of samples across various categories and computes the weighted accuracy for each category based on its sample proportion to acquire the overall accuracy. The calculation formula is as follows [118]:

(3) $$\mathrm{ACC}=\frac{1}{N}\sum_{i=1}^{N}w_{i}\cdot \frac{TP_{i}+TN_{i}}{TP_{i}+TN_{i}+FP_{i}+FN_{i}}$$

where $w_{i}$ is the weight of class $i,TP_{i}$ is the number of true positives in class $i,TN_{i}$ is the number of true negatives in class $i,FP_{i}$ is the number of false positives in class *i*, and $FN_{i}$ is the number of false negatives in class *i*.

#### 5.1.2. Unweighted Average Accuracy (UACC):

The UACC is calculated by computing the mean of accuracies for different emotion categories while disregarding any imbalances between these categories. The following formula represents the calculation process [118]:

(4) $$\mathrm{UACC}=\frac{1}{N}\sum_{i=1}^{N}\frac{TP_{i}+TN_{i}}{TP_{i}+TN_{i}+FP_{i}+FN_{i}}$$

#### 5.1.3. Weighted Average F1 (F1)

The F1 score is a widely employed evaluation metric in the field of emotion classification. It is calculated by combining precision and recall according to the formula provided below [118]:

(5) $$F1=\frac{1}{N}\sum_{i=1}^{N}w_{i}\cdot \frac{2\cdot {\mathrm{Precision}}_{i}\cdot {\mathrm{Recall}}_{i}}{{\mathrm{Precision}}_{i}+{\mathrm{Recall}}_{i}}$$

where Precision ${}_{i}=\frac{TP_{i}}{TP_{i}+FP_{i}}$ is the precision of class *i*, and Recall $i=\frac{TP_{i}}{TP_{i}+FN_{i}}$ is the recall of class *i*.

#### 5.1.4. Unweighted Average F1 (UF1)

The UF1 score computes the arithmetic average of F1 scores for each emotion category. Its calculation formula is shown below [118].

(6) $$\mathrm{UF}1=\frac{1}{N}\sum_{i=1}^{N}\frac{2\cdot {\mathrm{Precision}}_{i}\cdot {\mathrm{Recall}}_{i}}{{\mathrm{Precision}}_{i}+{\mathrm{Recall}}_{i}}$$

### 5.2. Regression Evaluation Metrics

#### 5.2.1. Mean Squared Error (MSE)

The MSE is a commonly employed evaluation metric for regression tasks. It calculates the average of the squared differences between the predicted values and the true labels. For continuous emotion prediction tasks, a lower MSE signifies that the model’s predictions align more closely with the true emotion values [119].

#### 5.2.2. Root Mean Squared Error (RMSE)

The RMSE is obtained by calculating the square root of the MSE. A lower RMSE indicates fewer prediction errors in the model. The calculation process is outlined as follows [119]:

(7) $$\mathrm{RMSE}=\sqrt{\mathrm{MSE}}$$

#### 5.2.3. Pearson Correlation Coefficient (PCC)

The PCC quantifies the linear relationship between the predicted values of a model and the actual emotional values. PCC values fall within the range of −1 to 1, where values near 1 signify a strong positive correlation, values near −1 denote a significant negative correlation, and values near 0 signify no correlation. The PCC is calculated as follows [120]:

(8) $$\mathrm{PCC}=\frac{\sum_{i=1}^{N}\left(y_{i}-\mu_{y}\right)\left({\hat{y}}_{i}-\mu_{\hat{y}}\right)}{\sqrt{\sum_{i=1}^{M}{\left(y_{i}-\mu_{y}\right)}^{2}\cdot \sum_{i=1}^{M}{\left({\hat{y}}_{i}-\mu_{\hat{y}}\right)}^{2}}}$$

where $\mu_{y}$ and $\mu_{\hat{y}}$ are the means of actual and predicted emotion scores.

#### 5.2.4. Concordance Correlation Coefficient (CCC)

The CCC combines the advantages of PCC and MSE. It not only captures the co-variation relationship between predictions and ground truth but also reflects its deviation. Therefore, it provides a better reflection of the alignment between predictions and ground truth, making it a widely utilized performance evaluation metric for continuous-dimensional sentiment prediction. Higher CCC values indicate strong performance in terms of consistency and accuracy. The specific calculation process for CCC is as follows [119]:

(9) $$\mathrm{CCC}=\frac{2\cdot \rho \cdot \sigma_{y}\cdot \sigma_{\hat{y}}}{\sigma_{y}^{2}+\sigma_{\hat{y}}^{2}+{\left(\mu_{y}-\mu_{\hat{y}}\right)}^{2}}$$

where ρ is the Pearson correlation coefficient and $\sigma_{y}$ and $\sigma_{\hat{y}}$ are the standard deviations of actual and predicted emotion scores.

## 6. Analysis of Research Results

Multimodal emotion recognition aims to integrate diverse modalities, including voice, text, and facial expressions, to precisely recognize and classify emotions. Table 2 provides an overview of recent seminal studies in this domain, highlighting 17 advanced multimodal emotion recognition techniques based on features, fusion strategies, datasets, and evaluation metrics. An analysis of this table reveals the following insights:

- Vocally, most studies prefer feature extraction using the COVAREP and openSMILE tools, which are open-source and adept at extracting a wide range of voice-related features. However, there is a growing inclination towards deep learning-based feature extraction, as illustrated by the significant outcomes achieved by Siriwardhana and colleagues using the Wav2Vec approach on the IEMOCAP and MELD datasets.
- In the textual domain, initial studies primarily relied on word vector models such as word2vec and Glove. With the evolution of deep learning, recent methods show a preference for sophisticated models like BERT and RoBERTa, which excel at capturing textual contextual nuances.
- Techniques for extracting facial expression features are diverse, encompassing methods like 3D-CNN, FACET, and OpenFace. Additionally, some studies are delving into technologies such as DenseNet, MTCNN, and Fabnet.
- Concerning fusion strategies, Fine-grained Interaction Fusion remains a popular choice, owing to its ability to foster detailed interactions between modalities based on nuanced features.
- Regarding datasets, IEMOCAP emerges as the predominant choice, likely due to the accuracy of its emotion labels and its longstanding reputation. Nonetheless, emerging datasets like MELD and YouTube are gaining traction, possibly to explore emotion recognition challenges in multifaceted and authentic contexts.

In conclusion, the field of multimodal emotion recognition has witnessed remarkable progress in recent years, particularly in the realms of feature extraction and data fusion. By integrating diverse modalities, these techniques offer enhanced accuracy when it comes to understanding and representing human emotions, providing substantial support for applications in human–machine interaction and emotion surveillance.

## 7. Conclusions and Future Challenges

In this comprehensive survey, we have detailed the progression and current state of deep neural network-based MER technology. We have examined popular feature extraction methods, considered significant multimodal emotion databases, and evaluated the influence of deep learning technology in this rapidly developing field. Crucially, we have also explored representative fusion methods, such as early fusion, late fusion, hybrid fusion, and intermediate layer fusion, which are critical to the effective integration of modal information in deep neural networks. Despite these strides, numerous challenges persist in the field of MER, especially with regard to its application to deep learning.

- Scale, annotation, and diversity of datasets: Datasets are crucial to deep learning models’ performance and generalization ability. An ideal dataset should exhibit representativeness, diversity, and sufficient scale while maintaining high-quality labels. Datasets enable models to learn patterns through sample observation, while diverse data provide learning opportunities in different contexts. Large-scale datasets can mitigate overfitting issues, and high-quality labels can provide accurate supervision signals. Consequently, constructing a large-scale and diverse dataset becomes imperative in the MER domain. Nevertheless, the annotation of multimodal data requires professionals to subjectively evaluate text, speech, and images, which is both time-consuming and expensive. Thus, the construction of a challenging task lies in developing a high-quality, large-scale, and diverse MER dataset.
- Multimodal Fusion: The task of fusing data from disparate modalities for emotion recognition is another predicament. Temporal misalignment and heterogeneities among features across different modalities can make the fusion process complex. This complexity often leads to models not fully utilizing the supplementary information from various modalities, which in turn undermines their performance in emotion recognition. In this context, information theory and entropy offer profound insights. By quantifying the information content in various modalities through entropy, it becomes feasible to evaluate the uncertainty or predictability of each data source. This approach can pinpoint modalities that provide substantial information while also identifying those that introduce noise or redundancy. Additionally, employing information theory concepts, such as mutual information, can elucidate the extent of interdependence between modalities. Such insights would facilitate a more harmonious and informed fusion process, ensuring that the relationships and synergies between modalities are optimally leveraged. Therefore, harnessing information theory to craft a potent strategy for amalgamating textual, facial, and auditory features—and seamlessly incorporating this multimodal information into a comprehensive model—is of paramount importance.
- Generalization Ability of Models: Despite the proposal of numerous excellent MER models, they are usually trained on specific datasets that rely on non-realistic scenarios, making them difficult to adapt to industrial applications. Therefore, MER models need to possess good generalization capability to be applicable to different scenarios and tasks. However, due to limitations in datasets and the complexity of models, the generalization capability of models with regard to new domains or unseen data remains a challenge. Addressing the construction of more universal and transferable models is one of the challenges that must be tackled.
- Leveraging Large-scale Training and Computational Resources: To attain greater accuracy and improved outcomes, deep learning models commonly necessitate abundant data and computational resources for training purposes. Nevertheless, acquiring extensive annotated data and conducting model training in the context of MER presents a formidable and expensive endeavor. Furthermore, ample computational resources and storage space are indispensable for large-scale training. Consequently, effectively utilizing limited data and computational resources, as well as accelerating the training process, is a challenge that needs to be addressed.
- Ethical and Privacy Issues: MER is a technology that extensively analyzes various data modalities, including speech, text, and facial cues, to discern individuals’ emotional states. This in-depth analysis often involves the processing of highly personal and intimate details about a person’s emotions and experiences. In practical applications, this level of analysis results in a direct interaction with users’ private information, creating a significant risk of potential privacy breaches. Therefore, striking a balance between ensuring model performance, protecting user privacy, ensuring reasonable data usage, and safeguarding user rights is an important challenge.

In conclusion, while considerable strides have been made in the realm of deep neural network-based MER, many obstacles remain. Overcoming these challenges will not only push the boundaries of what is achievable in this field but will also enable more robust, efficient, and ethically sound systems for emotion recognition. The future of MER is one filled with exciting opportunities for advancement and innovation. As we move forward, the focus will be on creating solutions that can overcome these hurdles and pave the way for a new era in emotion recognition technology.

## Figures and Tables

**Table 1:** Statistics on popular multimodal emotion recognition datasets. “N/A” indicates that the corresponding reference does not provide the relevant value.

Dataset	Samples	Language	Type	Recording Environment	Emotions	Data Sources	Speakers
IEMOCAP (2008) [24]	10,039	English	Acted and Natural	In the lab	Discrete: anger, sadness, happiness, disgust, fear, surprise, frustration, excited, and neutral states; Continuous: VAD (activation, valence, and dominance)	Recored	10
Youtube dataset (2011) [25]	47	English	Natural	In the wild	Discrete: positive, negative, and neutral	Youtube	47
MOUD (2013) [26]	498	Spanish	Natural	In the wild	Discrete: positive, negative, and neutral	Youtube	80
ICT-MMMO (2013) [27]	370	English	Natural	In the wild	Discrete: strongly negative, weakly negative, neutral, weakly positive, and strongly positive	Youtube and ExpoTV	370
CMU-MOSI (2016) [28]	2199	English	Natural	In the wild	Discrete: positive, negative	Youtube	89
NNIME (2017) [29]	6701	Chinese	Natural	In the lab	Discrete: anger, sadness, happiness, frustration, neutral, and surprise; Continuous: VA (valence and arousal)	Record	44
CMU-MOSEI (2018) [30]	23,453	English	Natural	In the wild	Discrete: anger, happiness, disgust, sadness, fear, and surprise	Youtube	1000
OMG(2018) [31]	7371	English	Acted and Natural	In the wild	Discrete: anger, happiness, disgust, sadness, fear, surprise, and neutral; Continuous: VA (valence and arousal)	Youtube	N/A
MELD (2019) [32]	13,708	English	Acted	In the wild	Discrete: (1) joy, sadness, anger, fear, disgust, surprise, and neutral; (2) positive, negative, neutral	TV series Friends	407
SEWA (2019) [33]	2562	Chinese, English, German, Greek, Hungarian, and Serbian	Natural	In the wild	Continuous: VA (valence and arousal)	Record	398
CH-SIMS (2020) [34]	2281	Chinese	Acted	In the wild	Discrete: negative, weakly negative, neutral, weakly positive, and positive	Movies, TV series	N/A
CH-SIMS V2.0 (2022) [35]	4402	Chinese	Acted	In the wild	Discrete: negative, weakly negative, neutral, weakly positive, and positive	Movies, TV series, etc.	N/A

**Table 2:** Summary of the state of the art in Multimodal Emotion Recognition. Here, S represents speech, T represents text, and F represents facial expressions.

Method	Feature Extraction	Fusion Method	Datasets	Performance
Poria et al. [121]	S: openSMILE T: word2vec F: 3D-CNN	Early Fusion	CMU-MOSI	ACC: 0.813 (2 classes)
Liu et al. [100]	S: COVAREP T: Glove F: FACET	Utterance-level Interaction Fusion	IEMOCAP	UF1: 0.831 (4 classes)
Zadeh et al. [111]	S: COVAREP T: Glove F: FACET	Fine-grained Interaction Fusion	a. Youtube b. MOUD c. IEMOCAP	a. ACC: 0.610, F1: 0.607 (3 classes); b. ACC: 0.811, F1: 0.804 (2 classes); c. ACC: 0.365, F1: 0.349 (9 classes)
Pham et al. [122]	S: MFCC T: Glove F: FACET and OpenFace	Fine-grained Interaction Fusion	CMU-MOSI	ACC: 0.765, F1: 0.734 (2 classes)
Poria et al. [123]	S: openSMILE T: CNN F: 3D-CNN	Early Fusion	a. IEMOCAP; b. MOUD; c. CMU-MOSI;	a. ACC: 0.716 (4 classes); b. ACC: 0.679 (2 classes); c. ACC: 0.767 (2 classes)
Zadeh et al. [30]	S: COVARE T: Glove F: MTCNN [124]	Fine-grained Interaction Fusion	CMU-MOSEI	UACC: 0.624, UF1: 0.763 (6 classes)
Majumber et al. [125]	S: openSMILE T: word2vec F: 3D-CNN	Fine-grained Interaction Fusion	a. CMU-MOSI b. IEMOCAP	a. ACC: 0.800 (2 classes); b. ACC: 0.765 (4 classes)
Tsai et al. [126]	S: COVAREP T: Glove F: FACET	Fine-grained Interaction Fusion	a. ICT-MMMO b. Youtube c. MOUD d.IEMOCAP	a. ACC: 0.813, F1: 0.792 (2 classes); b. ACC: 0.533, F1: 0.524 (3 classes); c. ACC: 0.821, F1: 0.817 (2 classes); d.UACC: 0.848, UF1: 0.814 (6 classes)
Wang et al. [127]	S: COVAREP T: Glove F: FACET	Fine-grained Interaction Fusion	IEMOCAP	UACC: 0.819, UF1: 0.812 (4 classes)
Tsai et al. [114]	S: COVAREP T: Glove F: FACET	Fine-grained Interaction Fusion	IEMOCAP	UACC: 0.747, UF1: 0.715 (4 classes)
Pham et al. [128]	S: COVAREP T: Glove F: FACET	Fine-grained Interaction Fusion	a. ICT-MMMO b. Youtube	a. ACC: 0.813, F1: 0.808 (2 classes); b. ACC: 0.517, F1: 0.524 (2 classes)
Liang et al. [94]	S: OpenSMILE T: BERT F: DenseNet [129]	Simple Concatenation Fusion	a. IEMOCAP b. MELD	a. ACC: 0.756, UACC: 0.745 (4 class); b. F1: 0.571 (7 classes)
Mittal et al. [112]	S: 12 MFCC, pitch, voiced/unvoiced segmentation features, glottal source parameters, among others. T: Glove F: Facial recognition models, facial action units, and facial landmarks.	Fine-grained Interaction Fusion	a. IEMOCAP b. CMU-MOSEI	a. ACC:0.827, F1: 0.824 (4 classes); b. ACC: 0.890, F1: 0.802 (6 classes)
Wang et al. [130]	S: OpenSMILE T: CNN F: 3D-CNN	Fine-grained Interaction Fusion	a. IEMOCAP b. CUM-MOSI c. MELD d.CMU-MOSI	a. ACC: 0.608 (6 classes); b. ACC: 0.827 (2 classes); c. ACC:0.620 (7 classes); d.ACC:0.827 (2 classes)
Sun et al. [131]	S: COVAREP T: BERT F: FACET	Fine-grained Interaction Fusion	IEMOCAP	UACC: 0.830, UF1: 0.818 (4 classes)
Siriwardhana et al. [132]	S: Wav2Vec T: RoBERTa F: Fabnet [133]	Fine-grained Interaction Fusion	a. IEMOCAP b. MELD	a. UACC: 0.847 UF1: 0.842 (4 classes); b. ACC: 0.643, F1: 0.639 (7 classes)
Lv et al. [116]	S: COVAREP T: BERT F: FACET	Fine-grained Interaction Fusion	a. IEMOCAP b. CMU-MOSI	a. UACC: 0.851, UF1: 0.838 (4 classes); b. ACC: 0.836, F1: 0.834 (2 classes)

## Data Availability

Not applicable.
